# Cell-specific expression of aquaporin-5 (*Aqp5*) in alveolar epithelium is directed by GATA6/Sp1 via histone acetylation

**DOI:** 10.1038/s41598-017-03152-7

**Published:** 2017-06-14

**Authors:** Per Flodby, Changgong Li, Yixin Liu, Hongjun Wang, Megan E. Rieger, Parviz Minoo, Edward D. Crandall, David K. Ann, Zea Borok, Beiyun Zhou

**Affiliations:** 1 Will Rogers Institute Pulmonary Research Center, Division of Pulmonary, Critical Care and Sleep Medicine, Department of Medicine, University of Southern California, Los Angeles, California USA; 2 Division of Neonatology, Department of Pediatrics, University of Southern California, Los Angeles, California USA; 3 Department of Pathology, University of Southern California, Los Angeles, California USA; 4 Department of Biochemistry and Molecular Biology, University of Southern California, Los Angeles, California USA; 5 Norris Comprehensive Cancer Center, Keck School of Medicine, University of Southern California, Los Angeles, California USA; 6 Mork Family Department of Chemical Engineering and Materials Science, Viterbi School of Engineering, University of Southern California, Los Angeles, California USA; 7 Department of Diabetes & Metabolic Diseases, Diabetes & Metabolism Research Institute, Beckman Research Institute, City of Hope Medical Center, Duarte, California USA

## Abstract

Epigenetic regulation of differentiation-related genes is poorly understood. We previously reported that transcription factors GATA6 and Sp1 interact with and activate the rat proximal 358-bp promoter/enhancer (p358P/E) of lung alveolar epithelial type I (AT1) cell-specific gene aquaporin-5 (*Aqp5*). In this study, we found that histone deacetylase (HDAC) inhibitor suberoylanilide hydroxamic acid (SAHA) increased AQP5 expression and Sp1-mediated transcription of p358P/E. HDAC3 overexpression inhibited Sp1-mediated *Aqp5* activation, while HDAC3 knockdown augmented AQP5 protein expression. Knockdown of GATA6 or transcriptional co-activator/histone acetyltransferase p300 decreased AQP5 expression, while p300 overexpression enhanced p358P/E activation by GATA6 and Sp1. GATA6 overexpression, SAHA treatment or HDAC3 knockdown increased histone H3 (H3) but not histone H4 (H4) acetylation within the homologous p358P/E region of mouse *Aqp5*. HDAC3 binds to Sp1 and HDAC3 knockdown increased interaction of GATA6/Sp1, GATA6/p300 and Sp1/p300. These results indicate that GATA6 and HDAC3 control *Aqp5* transcription via modulation of H3 acetylation/deacetylation, respectively, through competition for binding to Sp1, and suggest that p300 modulates acetylation and/or interacts with GATA6/Sp1 to regulate *Aqp5* transcription. Cooperative interactions among transcription factors and histone modifications regulate *Aqp5* expression during alveolar epithelial cell transdifferentiation, suggesting that HDAC inhibitors may enhance repair by promoting acquisition of AT1 cell phenotype.

## Introduction

The alveolar epithelium lining the internal surface of the lung is comprised of two cell types, alveolar epithelial type I (AT1) and type II (AT2) cells, which have distinct morphology, molecular phenotypes and functions^1^. AT2 cells are cuboidal, synthesize surfactant proteins (SFTPA, SFTPB, SFTPC and SFTPD) and express the homeodomain transcription factor NKX2.1, while AT1 cells are squamous, cover the majority of the gas exchange surface of the lung and are identified by expression of specific phenotypic markers (e.g., water channel protein aquaporin-5 (AQP5), podoplanin/T1α, caveolin and receptor for advanced glycation end products (RAGE)). Studies in injury models^2–4^, analyses of *in vitro* primary AT2 cell cultures^5, 6^ and lineage tracing experiments^7–9^ have provided strong evidence that AT2 cells both self-renew and transdifferentiate into AT1 cells during alveolar epithelial maintenance and following injury. *In vitro* studies have further demonstrated that AT2 cells in primary culture that have already acquired AT1 cell-like phenotypic properties can re-express AT2 cell markers under certain culture conditions^10–14^, while *in vivo* permanently labeled HOPX-expressing AT1 cells are able to express AT2 cell markers in response to partial pneumonectomy^15^, indicating considerable phenotypic plasticity. Elucidation of molecular mechanisms that regulate cell-specific gene expression accompanying phenotypic transitions between AT2 and AT1 cells is important to understand how alveolar epithelial cells (AEC) are maintained and regenerate following injury.

Epigenetic modulation has been implicated in transcriptional regulation of tissue-specific gene expression and cell differentiation in eukaryotic cells, but evaluation of these mechanisms in the context of AT2 and AT1 cell-specific gene expression has been limited. In this regard, interaction of the homeodomain transcription factor NKX2.1 (also known as thyroid transcription factor-1 (TTF1)) with ATP-dependent chromatin remodeling protein BRG1 at the unmethylated *Sftpb* promoter increased *Sftpb* expression, which correlated with increased H3K4 trimethylation. These observations suggest that NKX2.1 cooperates with proteins mediating both DNA methylation and histone modification to regulate *Sftpb* ^16^. In fetal lung, *Sftpa* activation by cyclic AMP (cAMP) and cytokines (e.g., interleukin-1α (IL-1α)) is mediated by increased NKX2.1, CREB (cAMP-response element-binding protein)-binding protein (CBP) and NF-κB binding to the TTF-1-binding element (TBE) of the *Sftpa* promoter, which is correlated with increased H3K9 acetylation, decreased dimethylation of H3K9 and decreased histone deacetylase (HDAC) binding to the TBE. In contrast, *Sftpa* inhibition by glucocorticoids is associated with enhanced glucocorticoid receptor (GR) and HDAC binding to the promoter and decreased H3 acetylation^17, 18^. Additionally, activation of T1α is regulated by methylation at a key Sp1 site in the proximal promoter^19^.

AQP5 is a water channel protein that is highly expressed in lung, salivary gland and lacrimal gland^20, 21^. In the rat lung, AQP5 is exclusively expressed on the apical membrane of AT1 (and not AT2) cells^20, 22^. AQP5 expression in human salivary gland is regulated by acetylation of histone H4^23^ and DNA methylation^24^. In rat lung, activation of *Aqp5* in AT1 cells is associated with increased Sp1 binding to the hypomethylated proximal *Aqp5* promoter^25^. However, a role for histone modifications in regulation of *Aqp5* expression in AEC has not been reported. We previously showed that AQP5 expression is controlled by the zinc-finger transcription factor GATA6, involving interactions with Sp1 at the rat 358 bp proximal promoter/enhancer region (p358P/E)^26^. In the current study, we examined the contributions of histone modifications to GATA6/Sp1-mediated *Aqp5* transactivation. Our results demonstrate that GATA6 increases H3 acetylation, while HDAC3 causes H3 deacetylation at the proximal *Aqp5* promoter/enhancer, leading to activation and repression of *Aqp5* transcription, respectively. These data suggest that epigenetic regulation of *Aqp5* transcription involves competition of GATA6 and HDAC3 for binding to Sp1. Furthermore, we demonstrate a role for p300 in GATA6/Sp1 activation of *Aqp5* transcription, possibly via modulation of H3 acetylation and/or GATA6 and Sp1 activity.

## Results

### Histone acetylation/deacetylation is involved in *Aqp5* gene regulation

**Figure 1:**
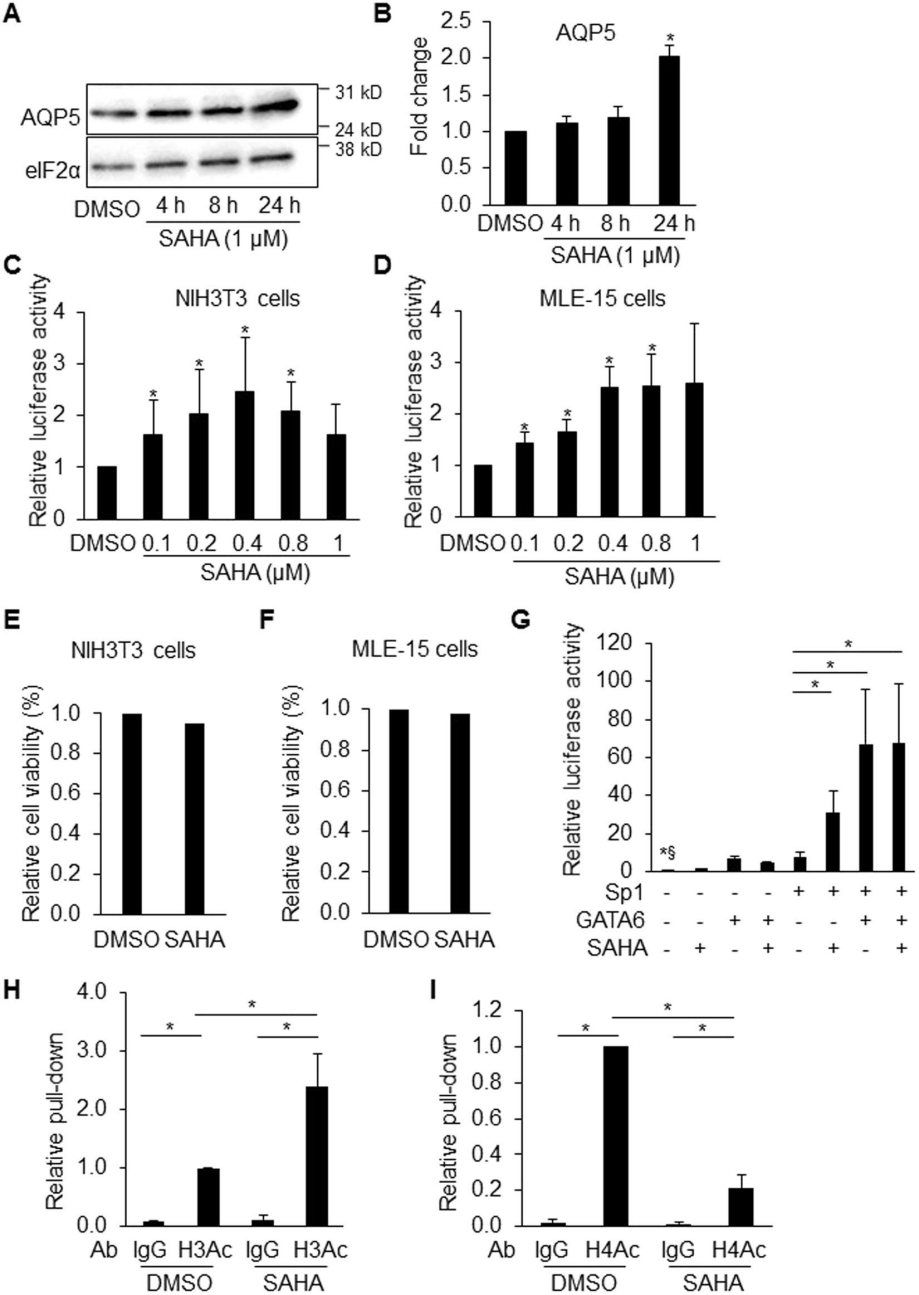
HDAC inhibitor increases AQP5 expression. Representative western blot (WB) (**A**) and corresponding quantitative analysis (**B**) shows that treatment of MLE-15 cells with HDAC inhibitor SAHA for 24 h increases AQP5 (27 kD) protein expression (normalized to eIF-2α (36 kD)) (n = 4, *p < 0.05 compared to DMSO). SAHA increases *Aqp5* luciferase activity in a dose-dependent manner in both NIH3T3 (**C**) and MLE-15 cells (**D**) transfected with -358-*Aqp5*-Luc following 18 h treatment. Protein concentrations were used for sample normalization (n = 3, *p < 0.05 compared to DMSO). Twenty-four h of SAHA (1 µM) treatment had no cytotoxic effects on NIH3T3 (**E**) and MLE-15 cells (**F**). (**G**) GATA6 (pCDNA3/GATA6), Sp1 (pCMV/Sp1) and their corresponding empty vectors were transfected individually or in combination into NIH3T3 cells, followed by treatment with SAHA (0.8 µM) for 18 h. SAHA significantly increases Sp1-activated -358 *Aqp5* promoter/enhancer activity. Protein concentrations were used for sample normalization. n = 3 except for column 2 (empty vectors with SAHA), where n = 2, *^§^p < 0.05 shows significant difference between control (column 1, empty vectors with DMSO) and all other groups except column 2. Since column 2 is only n = 2, we did not perform statistical analysis for comparison with control. *p < 0.05 compared to column 5 (Sp1 with DMSO). ChIP with anti-acetyl-H3 (H3Ac) and anti-acetyl-H4 (H4Ac) Abs demonstrates enrichment of H3 acetylation (**H**) and decreased H4 acetylation (**I**) at the *Aqp5* promoter/enhancer region homologous to the proximal 358-bp of the rat *Aqp5* promoter following SAHA treatment (1 µM, 24 h) in MLE-15 cells (n = 3, *p < 0.05). ChIP efficiency was calculated relative to untreated cells precipitated with H3Ac and H4Ac Ab, respectively, which was set as 1. Rabbit IgG pull-down is used as control.

We previously identified a rat proximal *Aqp5* promoter/enhancer fragment, p358P/E (−358 bp relative to the transcription start site (TSS)), containing multiple putative Sp1 sites, and showed that GATA6 activates this region via interactions with Sp1^26^. Since Sp1 binds to both p300, which has histone acetylase activity, and HDACs^27–29^, we hypothesized that modification of histone acetylation is involved in GATA6/Sp1-mediated *Aqp5* gene expression. To test this hypothesis, mouse lung epithelial (MLE-15) cells were treated with HDAC inhibitor SAHA. As shown in a representative western blot (Fig. 1A), SAHA (1 µM) increased AQP5 protein expression in MLE-15 cells by 203 ± 14.7% at 24 h (Fig. 1B). To further investigate whether SAHA regulates *Aqp5* expression at the transcriptional level, we performed transient transfection assays in MLE-15 and NIH3T3 cells in which GATA6 and Sp1 have been shown to regulate *Aqp5* transcriptional activity^26^. In both MLE-15 and NIH3T3 cells, SAHA increased p358P/E transcriptional activity in a dose-dependent manner (Fig. 1C,D), implicating histone acetylation/deacetylation in regulation of AQP5 expression, although luciferase (Luc) activity declined at higher doses in NIH3T3 cells. Cell viability assays showed no toxicity of SAHA (1 µM) treatment for 24 h in both NIH3T3 and MLE-15 cells (Fig. 1E,F). Furthermore, SAHA significantly increased Sp1-mediated -358-*Aqp5*-Luc promoter activity in NIH3T3 cells (Fig. 1G), suggesting that Sp1 binding with HDACs at the p358P/E is involved in responses to SAHA. SAHA did not further increase GATA6- or GATA6/Sp1-mediated activation (Fig. 1G), perhaps because either GATA6 binding to Sp1 interferes with binding of HDACs to Sp1 or GATA6/Sp1 binding inhibited HDAC activity^30, 31^. To further investigate if SAHA treatment affects histone acetylation at the *Aqp5* promoter/enhancer region, we performed qPCR following chromatin immunoprecipitation (ChIP) in MLE-15 cells treated with SAHA using antibodies against the N-terminal portion of acetylated H3 and H4. There was enrichment of H3 acetylation (Fig. 1H) but decreased H4 acetylation (Fig. 1I) at the *Aqp5* promoter/enhancer region homologous to rat p358P/E compared to DMSO control, indicating that SAHA induction of *Aqp5* expression involves increased H3 acetylation (but decreased H4 acetylation) at the proximal *Aqp5* promoter/enhancer region.

### HDAC3 inhibits *Aqp5* gene expression

**Figure 2:**
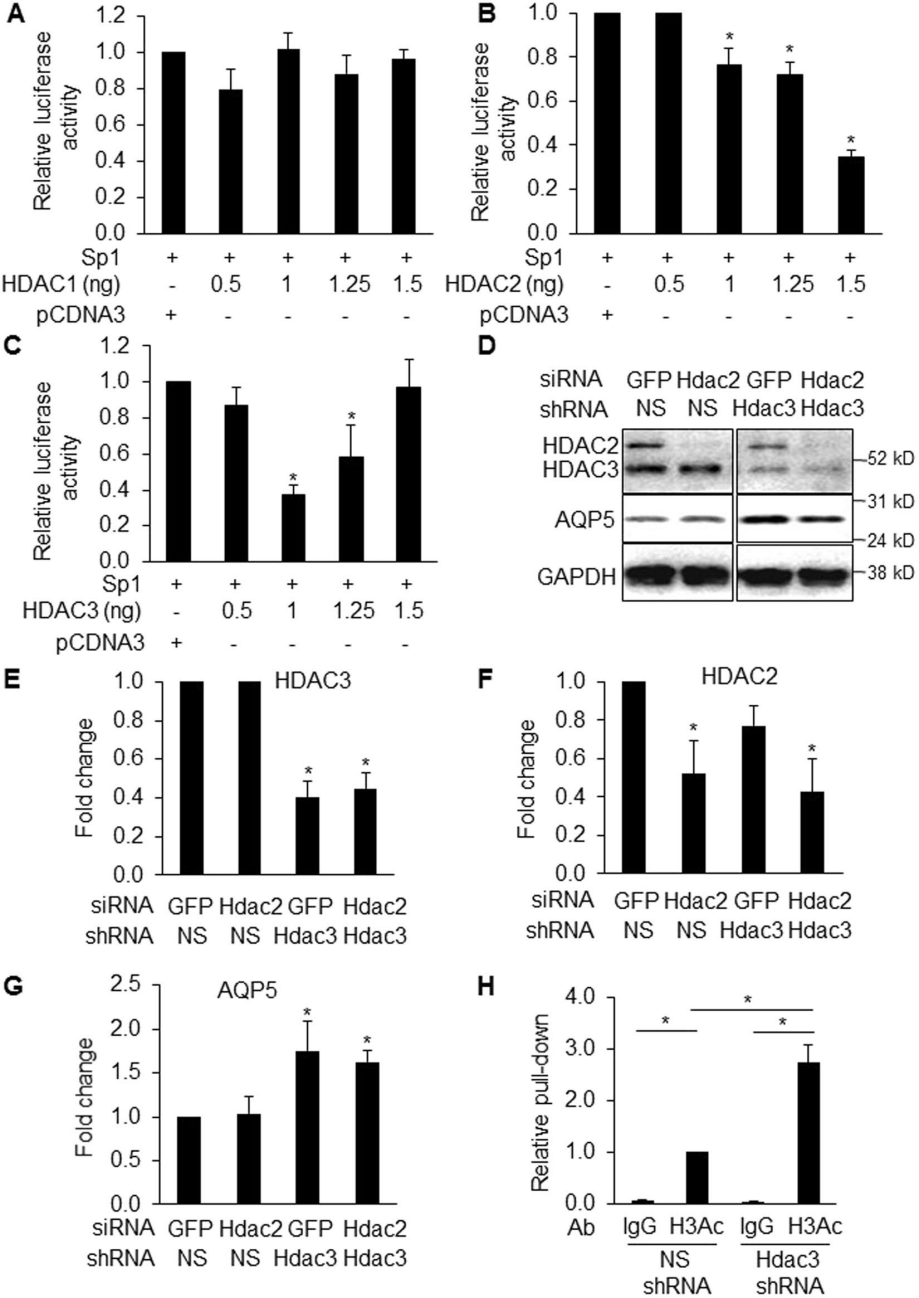
HDAC3 modulates expression of AQP5. NIH3T3 cells were co-transfected with 358-*Aqp5*-Luc, Sp1 and increasing amounts of HDAC1 (**A**), HDAC2 (**B**) and HDAC3 (**C**) expression vectors or empty vector (pCDNA3). HDAC2 and HDAC3, but not HDAC1 inhibited Sp1-activated *Aqp5* transcription. Protein concentrations were used for sample normalization (n = 3, *p < 0.05 compared to empty vector). WB (**D**) and corresponding quantitative analysis (**E**–**G**) show that AQP5 expression (normalized to GAPDH (37 kD)) is significantly upregulated (**D**,**G**) following knockdown of HDAC3 (59 kD) with shRNA (**D**,**E**) but not HDAC2 (49 kD) with siRNA (**D**,**F**) (n = 3, *p < 0.05 compared to non-silencing shRNA and GFP siRNA) in MLE-15 cells. (**H**) ChIP with anti-acetyl-H3 Ab (H3Ac) demonstrates enrichment of H3 acetylation at the *Aqp5* promoter/enhancer region homologous to the proximal 358-bp of the rat *Aqp5* promoter following HDAC3 knockdown in MLE-15 cells (n = 3, *p < 0.05). ChIP efficiency was calculated relative to non-silencing shRNA (NS-shRNA)-treated cells precipitated with H3Ac Ab, which was set as 1. Rabbit IgG pull-down is used as control.

Sp1 has been shown to interact with HDAC1, 2 and 3 in regulation of gene expression^27, 29, 32^. To investigate which HDAC is involved in regulation of Sp1 activation of *Aqp5* promoter/enhancer activity, -358-*Aqp5*-Luc was co-transfected with Sp1 and increasing amounts of HDAC1, 2 and 3 expression vectors or empty pCDNA3 vector in NIH3T3 cells. HDAC2 (Fig. 2B) and HDAC3 (Fig. 2C), but not HDAC1 (Fig. 2A), inhibited Sp1-activated -358-*Aqp5*-Luc transcription. However, at higher doses, HDAC3 did not show an inhibitory effect, possibly due to HDCA3 inhibition of an unknown repressor of *Aqp5* at higher concentrations or dependence of HDAC3 activity on its interaction with a corepressor (e.g., SMRT) or a serine/threonine protein phosphatase, PP4^33^. We then knocked down HDAC2 and HDAC3 alone or in combination (Fig. 2D) using *Hdac2* small interfering RNA (siRNA) and *Hdac3* short hairpin RNA (shRNA). Knockdown of HDAC3 (Fig. 2E) but not HDAC2 (Fig. 2F) increased AQP5 protein expression (Fig. 2G). These data suggest that, while both HDAC2 and HDAC3 can inhibit *Aqp5* transcription, compensatory effects from other HDACs following HDAC2 reduction likely exist. Additionally, HDAC2 may increase translation of some proteins by promoting sumoylation of eukaryotic translation initiation factor 4E (eIF4E), independently of its deacetylase activity^34^ which might in turn affect translation and therefore levels of AQP5 protein, resulting in no net change in AQP5 protein expression. To examine whether HDAC3 inhibition of *Aqp5* transcription is associated with H3 deacetylation at the −358 bp proximal *Aqp5* promoter/enhancer region, ChIP was performed using chromatin harvested from MLE-15 cells with/without HDAC3 knockdown, followed by pull-down with anti-acetyl-H3 antibody (Ab). qPCR following ChIP demonstrates enrichment of H3 acetylation at the *Aqp5* promoter/enhancer region in cells with HDAC3 knockdown compared to control shRNA (Fig. 2H), confirming that HDAC3 regulation of *Aqp5* expression involves changes in H3 acetylation at the proximal *Aqp5* promoter/enhancer region.

### GATA6 activates *Aqp5* gene expression concomitant with increased H3 acetylation

**Figure 3:**
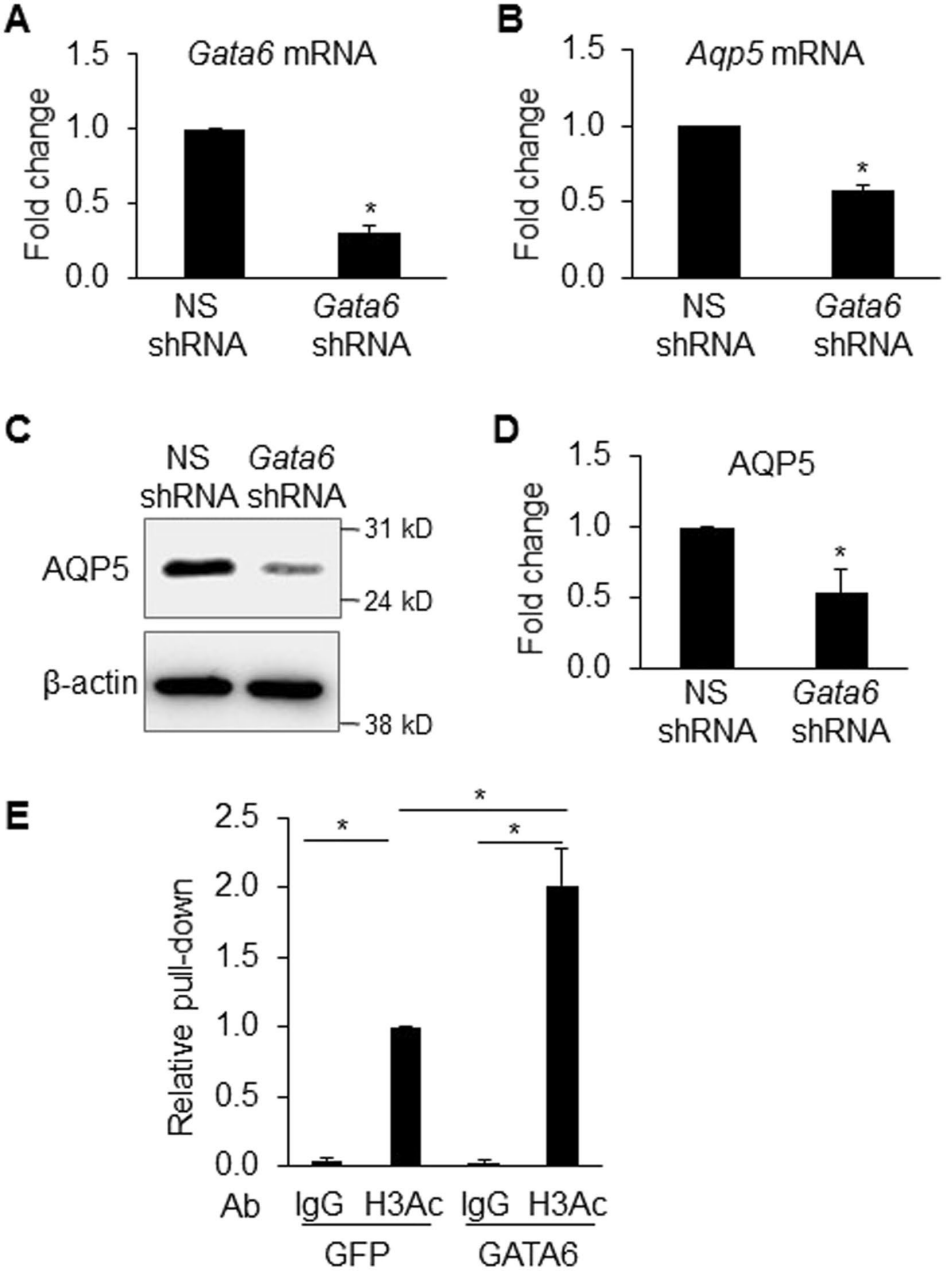
GATA6 regulation of AQP5 expression involves H3 acetylation. RNA and protein were harvested from MLE-15 cells transduced with lentivirus expressing *Gata6* or control non-silencing shRNA (MOI = 2) for 3 days. qRT-PCR shows that knockdown of *Gata6* (**A**) significantly decreases *Aqp5* mRNA (**B**) and protein (AQP5 normalized to β-actin (42 kD)) (**C**,**D**) expression (n = 3, *p < 0.05 compared to non-silencing shRNA (NS-shRNA)). (**E**) ChIP with anti-acetyl-H3 Ab (H3Ac) demonstrates enrichment of H3 acetylation at the *Aqp5* promoter/enhancer region homologous to the proximal 358-bp of the rat *Aqp5* promoter using chromatin harvested from MLE-15 cells transduced with GATA6-expressing lentivirus or control (GFP) (MOI = 2) (n = 3, *p < 0.05). ChIP efficiency was calculated relative to control virus-transduced cells precipitated with H3Ac Ab, which was set as 1. Rabbit IgG pull-down is used as control.

To further investigate the role of GATA6 in regulation of *Aqp5* transcription, we performed GATA6 knockdown in MLE-15 cells by transducing with lentivirus expressing *Gata6* or control non-silencing shRNA. Both qRT-PCR and western analysis confirmed that knockdown of *Gata6* (Fig. 3A) significantly decreases *Aqp5* mRNA (Fig. 3B) and AQP5 protein expression (Fig. 3C,D). Additionally, ChIP with anti-acetyl-H3 Ab demonstrated enrichment of H3 acetylation at the *Aqp5* promoter/enhancer region following GATA6 overexpression compared to control (Fig. 3E). These data suggest that increased H3 acetylation at the proximal *Aqp5* promoter/enhancer contributes to GATA6-mediated activation of *Aqp5* transcription. Precise mechanisms whereby GATA6 modulates H3 acetylation are unknown but, since SAHA does not affect GATA-6 activation of *Aqp5*-Luc, include the possibilities that GATA6 interaction with Sp1 decreases HDAC3 binding to Sp1 and/or GATA6 binding to the Sp1/HDAC3 complex inhibits HDAC3 activity. In addition, as shown further below, recruitment of transcriptional coactivators with histone acetyltransferase (HAT) activity (e.g., p300) may modulate acetylation.

### p300 but not CBP enhances GATA6/Sp1-mediated *Aqp5* transcription

**Figure 4:**
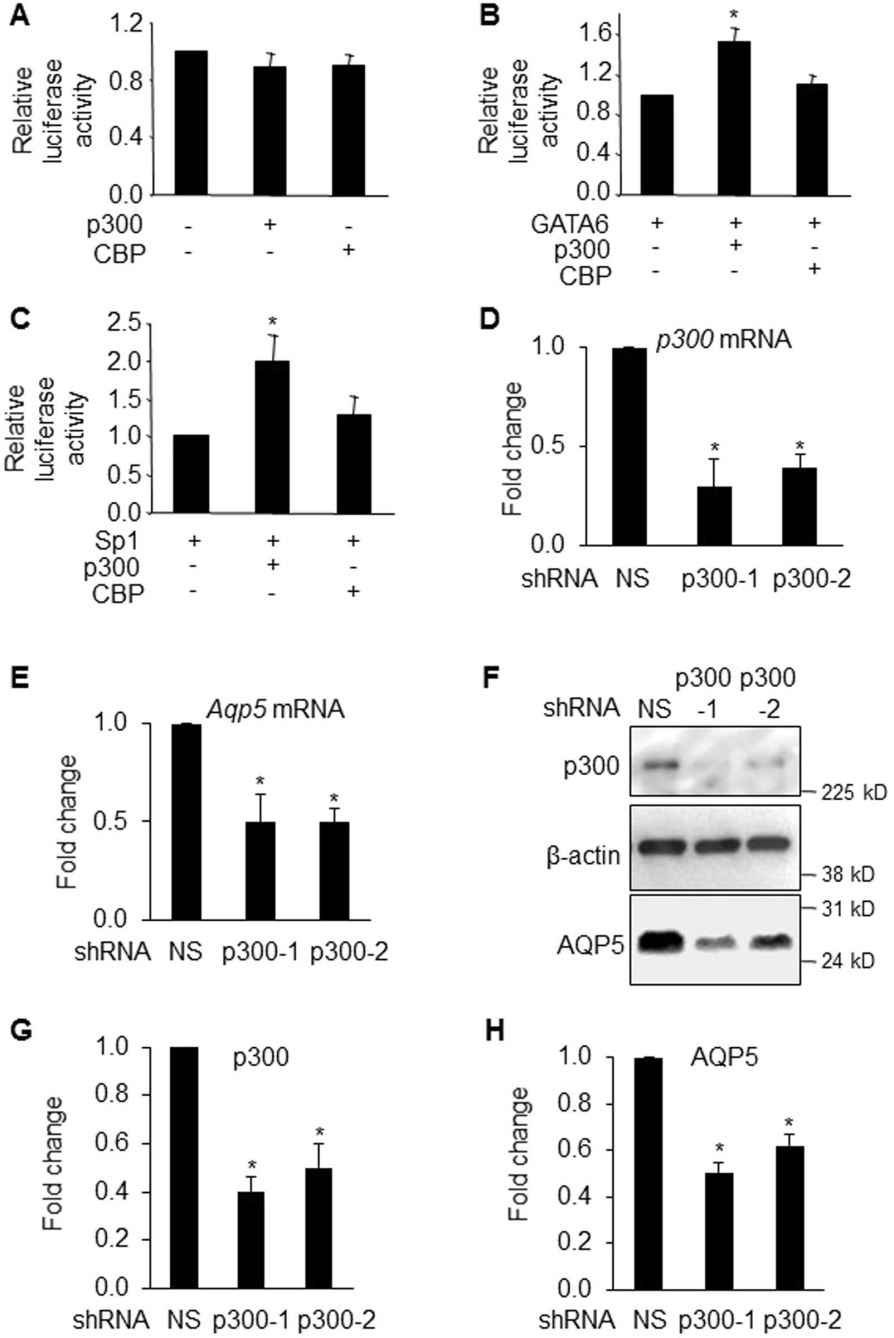
p300 increases GATA6/Sp1-mediated -358-*Aqp5*-Luc activity. (**A**–**C**) NIH3T3 cells were co-transfected with -358-*Aqp5*-Luc and different combinations of GATA6, Sp1, p300 and CBP expression vectors. Luciferase assays were performed 48 h after transfection. p300 and CBP have no effect on *Aqp5*-Luc activity (**A**), while p300 (but not CBP) augments both GATA6- (**B**) and Sp1- (**C**) mediated *Aqp5* transcriptional activity. Protein concentration was used to normalize samples for transfection efficiency (n = 3, *p < 0.05 compared to GATA6 or Sp1 only). qRT-PCR shows knockdown of *p300* mRNA using two different shRNAs (V2LMN_102133 indicated as p300-1 and V2LMN_90586 indicated as p300-2) (**D**) decreases *Aqp5* mRNA expression following p300 knockdown (**E**) (n = 4, *p < 0.05 compared to non-silencing shRNA control (NS shRNA)). WB shows knockdown of p300 (**F**,**G**) decreases AQP5 protein expression (**F**,**H**). (n = 4, *p < 0.05 compared to NS shRNA control).

p300 and CBP are highly homologous transcriptional coactivators that possess intrinsic HAT activity^35, 36^. It has been reported that p300/CBP increase H3 acetylation at promoter binding sites for Sp1 in transforming growth factor-β (TGFβ) target genes p21 and plasminogen activator inhibitor-1 (PAI-1) to activate transcription in mesangial cells^37^. Transient transfections were performed in NIH3T3 cells to determine if p300 or CBP are involved in modulation of *Aqp5* transcription by GATA6/Sp1. Neither p300 nor CBP alone had an effect on p358P/E activity (Fig. 4A). However, p300 but not CBP further augmented GATA6- (Fig. 4B) and Sp1- (Fig. 4C) mediated transcriptional activation of *Aqp5*-Luc. Consistent with results showing that knockdown of p300 decreases AQP5 expression in rat AEC in primary culture^38^, knockdown of p300 in MLE-15 cells with shRNA (Fig. 4D,F,G) significantly decreased *Aqp5* mRNA (Fig. 4E) and AQP5 protein expression (Fig. 4F,H). These findings suggest that p300, either through its intrinsic HAT activity or function as a transcriptional co-activator, regulates *Aqp5* transactivation through interactions with GATA6 and/or Sp1, and suggest a mechanism whereby GATA6 may regulate acetylation of the *Aqp5* proximal promoter/enhancer via recruitment of p300.

### HDAC3 competes with GATA6 for binding to Sp1

**Figure 5:**
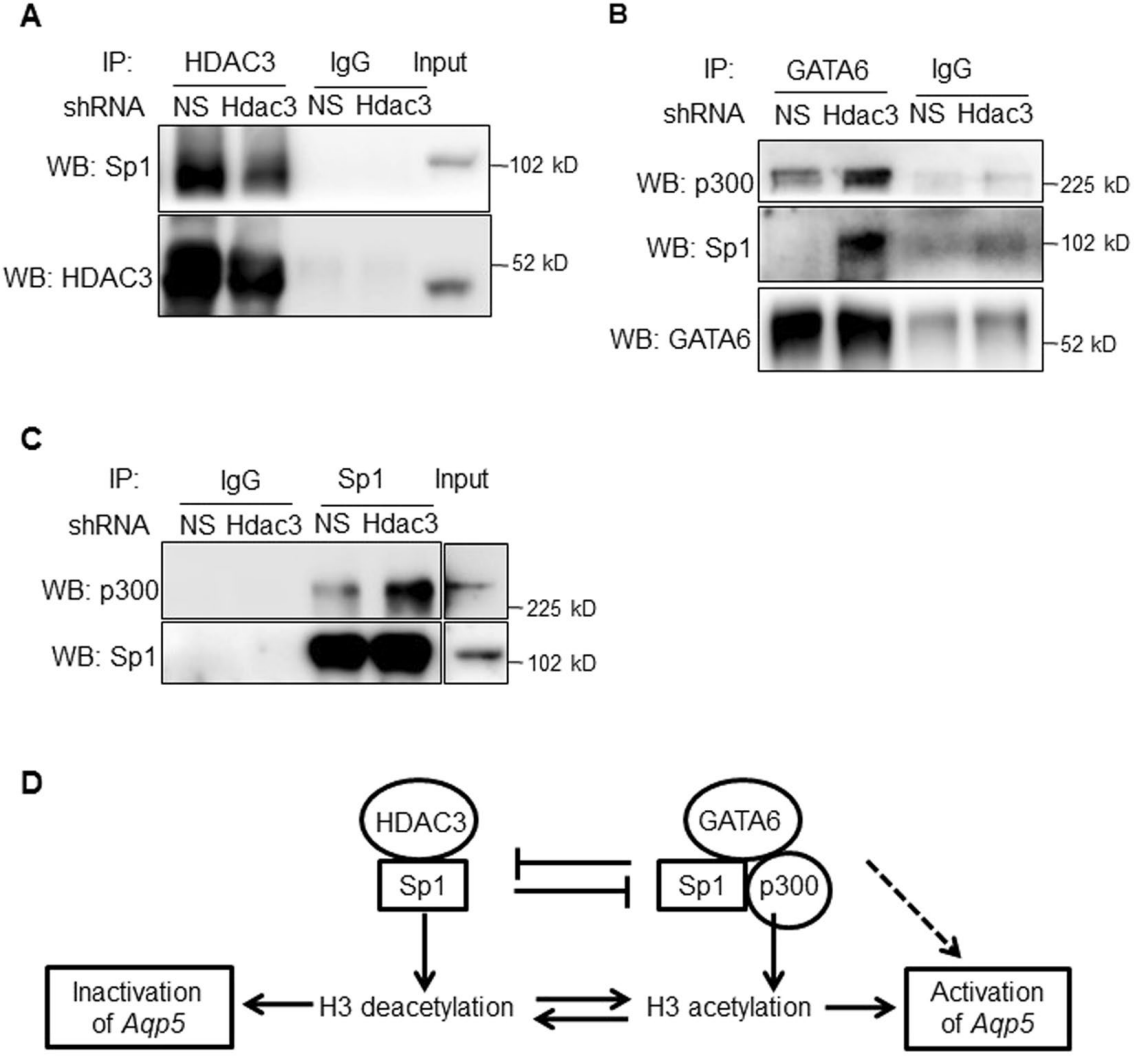
Knockdown of HDAC3 increases GATA6/Sp1/p300 interactions. Representative co-immunoprecipitation (co-IP) of cell lysate from MLE-15 cells transduced with *Hdac3* shRNA or non-silencing shRNA (NS) demonstrates decreased HDAC3/Sp1 interaction (**A**), increased GATA6/Sp1 and GATA6/p300 (**B**), and increased p300/Sp1 (**C**) interaction following HDAC3 knockdown. n = 3. (**D**) Model of Sp1-mediated *Aqp5* transcriptional regulation and involvement of HDAC3, GATA6, and p300: HDAC3 and GATA6 regulate Sp1-mediated *Aqp*5 transcription via H3 deacetylation and acetylation, respectively, at the proximal promoter/enhancer. Mechanisms underlying GATA6-dependent H3 acetylation at the *Aqp5* enhancer/promoter might involve competition between GATA6 and HDAC3 for binding to Sp1, as well as recruitment of histone acetylase p300 to the Sp1/GATA6 complex. In addition to effects on H3 acetylation, p300 might also modulate GATA6 and/or Sp1 activity.

To further examine if HDAC3 binds to Sp1 and if HDAC3 affects GATA6/Sp1 binding, co-immunoprecipitation (co-IP) was performed using protein lysates from MLE-15 cells with/without HDAC3 knockdown. As shown in Fig. 5A, HDAC3 binds to Sp1 as expected, and this interaction decreases following HDAC3 knockdown (Fig. 5A). HDAC3 knockdown also increased GATA6/Sp1 interaction (Fig. 5B), supporting the suggestion that HDAC3 competes with GATA6 for binding to Sp1. Additionally, co-IP showed that knockdown of HDAC3 increased the interaction of both GATA6 (Fig. 5B) and Sp1 (Fig. 5C) with p300, suggesting that GATA6 further recruits p300, perhaps due to increased affinity of the GATA6/Sp1 complex for p300. Based on the findings in this study, we suggest a model (Fig. 5D) where increased expression levels of GATA6 leads to increased GATA6/Sp1 interaction, decreased binding of HDAC3 to Sp1 and increased histone acetylation, contributing to *Aqp5* promoter activation. Furthermore, the findings indicate that p300, either through its intrinsic HAT activity and/or functioning as a transcriptional co-activator, regulates *Aqp5* transactivation through interactions with GATA6 and Sp1, and suggest a mechanism whereby GATA6 may regulate acetylation of the *Aqp5* proximal promoter/enhancer via recruitment of p300.

## Discussion

In the distal lung, AQP5 is specifically expressed in AT1 cells and plays an important role in alveolar homeostasis^21, 39, 40^. The role of epigenetic mechanisms, especially histone modifications, in regulation of AT1 cell-specific expression of *Aqp5* is not completely understood. In this study, we found that HDAC inhibitor SAHA increases expression of AQP5 and that HDAC3 inhibits Sp1-mediated transcriptional activation of *Aqp5*, which is associated with H3 acetylation/deacetylation, respectively, at the proximal promoter/enhancer. We demonstrated that GATA6 activation of *Aqp5* transcription involves H3 acetylation at the proximal promoter/enhancer and that p300 augments *Aqp5* activation by GATA6 and Sp1. We further provide evidence that HDAC3 binds to Sp1 and competes with GATA6/Sp1 binding, and that HDAC3 inhibition of *Aqp5* transcription is associated with H3 deacetylation. These data suggest that H3 acetylation/deacetylation is involved in regulation of differentiation-related *Aqp5* gene expression concomitant with GATA6/Sp1/p300 and/or Sp1/HDAC binding at the proximal promoter/enhancer. Although SAHA decreased H4 acetylation at the *Aqp5* promotor and a decrease in H4 acetylation is known to be associated with gene activation^41, 42^, the mechanisms underlying decreased H4 acetylation as a result of SAHA treatment and how this might contribute to increased *Aqp5* gene expression remain unknown and will require future studies. Nevertheless, our data are consistent with previous findings that the Sp family of transcription factors (e.g., Sp1 and Sp3) regulates cell-specific gene expression by recruiting HDACs or proteins with HAT activity (e.g., p300^43, 44^) to target gene promoters^37, 45^.

GATA6 is a key transcription factor that regulates organogenesis and epithelial cell differentiation in the lung^46^. GATA6 is the only GATA family member expressed in the distal epithelium of the developing lung, where it plays an important role in lung branching and AEC differentiation^46, 47^. GATA6 regulates expression of AT2 cell-specific genes (e.g., *Sftpc* ^48^, *Sftpa* ^49^ and *Nkx2*.*1* ^50^) while also regulating activity of the promoter/enhancer of the AT1 cell-specific gene *Aqp5* ^51^. Overexpression of dominant negative GATA6 in transgenic mice led to impaired AT1 cell development and reduced AQP5 expression *in vivo* ^51^. In our *in vitro* rat AEC culture model, expression of GATA6 increases concurrent with increases in AQP5 during AT2 to AT1 cell transdifferentiation. We have previously shown that GATA6 mediates activation of *Aqp5*, largely through interactions with Sp1 at the proximal promoter/enhancer region, further supporting a role for GATA6 in regulation of AT1 cell differentiation^26^. In this study, we demonstrate that GATA6 regulation of the AT1 cell-specific gene *Aqp*5 is associated with increased histone acetylation. Our data suggest that GATA6/Sp1 interaction alters acetylation by interfering with HDAC3/Sp1 binding and recruiting p300^43, 44^.

p300 is a transcriptional coactivator that does not directly bind to DNA. It encompasses different domains that enable p300 to regulate cell-specific gene expression by interacting with various transcription factors as an adaptor^52^. Additionally, the HAT domain of p300 catalyzes acetylation of promoter-bound histones, leading to chromatin opening and gene activation^36^. p300 can also acetylate transcription factors such as NF-κB^53^, Smad^37^, p53^54^ and Sp1^43, 44, 55^, as well as GATA family members GATA1^56^ and GATA4^57^, to modulate their transcriptional activity via changes in DNA binding activity, protein stability and interactions with other transcription factors. It will be important to elucidate if p300 mediates activation of *Aqp5* transcription by GATA6/Sp1 through histone acetylation, direct acetylation of GATA6/Sp1 or a combination thereof or independent of its acetylation activity. We propose a model (Fig. 5) in which GATA6 activates *Aqp5* transcription by interfering with HDAC3 binding to Sp1 leading to increased H3 acetylation, as well as recruitment of p300 that may acetylate H3 through its HAT activity and/or serve as a transcriptional coactivator.

Interestingly, p300 but not CBP is involved in GATA6/Sp1-mediated *Aqp5* transcription. Despite high homology between these coactivators, they differentially regulate cell function, with CBP/β-catenin interaction implicated in progenitor cell maintenance and p300/β-catenin promoting differentiation^58, 59^. We recently showed that p300, but not CBP, promotes AT2 to AT1 cell transdifferentiation through interaction with β-catenin^38^. Our data confirm previous findings that p300, but not CBP, plays a role in activation of AT1 cell-specific gene *Aqp5*. However, involvement of β-catenin in *Aqp5* gene activation mediated by the GATA6/Sp1/p300 complex reported here will require further investigation.

Our data strongly suggest that correct spatial and temporal regulation of *Aqp5* transcription via specific transcription factor interactions and histone modifications is important to maintain AT1 cell-specific gene expression. Altered epigenetic modifications and transcription factor binding on the *Aqp5* promoter may contribute to disease pathogenesis and impair AT1 cell recovery following injury. HDAC inhibitors may therefore be beneficial to promote re-epithelialization of the lung in response to injury.

## Methods

### Cell culture

NIH3T3 cells were grown at 37 °C in Dulbecco’s modified Eagle’s medium supplemented with 10% heat inactivated fetal bovine serum (FBS, Hyclone, Logan, Utah), penicillin (500 units/ml, Sigma-Aldrich, St. Louis, MO) and streptomycin (0.1 mg/ml, Sigma-Aldrich). MLE-15 cells (J. Whitsett, University of Cincinnati) were cultivated in HITES medium (RPMI-1640 medium supplemented with 10 nM hydrocortisone, 5 µg/ml insulin, 5 µg/ml human transferrin, 10 nM β-estradiol, 5 µg/ml selenium, 2 mM L-glutamine, 10 mM HEPES, 100 U/ml penicillin, 100 µg/ml streptomycin and 4% FBS (Atlanta Biologicals, Flowery Branch, GA).

### Transient transfection assays

MLE-15 or NIH3T3 cells were seeded at 6 × 10^4^ cells/well in 24-well plates one day before transfection. To examine the effect of SAHA on *Aqp5* transcription, MLE-15 or NIH3T3 cells were transfected with a 358-bp rat *Aqp*5-luciferase reporter (-358-*Aqp5*-Luc) construct^26^ using Superfect reagent (Qiagen, Valencia, CA). To examine the effect of SAHA on GATA6 and/or Sp1-mediated *Aqp5* transcription, -358-*Aqp5*-Luc was transfected into NIH3T3 cells, together with pCMV/Sp1 and/or pCDNA3/GATA6 expression vectors (or empty vector controls), individually or in combination. Four hours (h) after transfection, cells were treated with HDAC inhibitor suberoylanilide hydroxamic acid (SAHA) (#10009929, Cayman Chemical, Ann Arbor, Michigan) or DMSO as control. Cells were harvested after treatment with SAHA for 18 h for measurement of firefly luciferase activity (Promega, Madison, WI) that was normalized to protein concentration. To examine effects of HDAC1, HDAC2 and HDAC3 on *Aqp5* transcription, NIH3T3 cells were transfected with -358-*Aqp5*-Luc, together with varying amounts of pCDNA3/HDAC1, pCDNA3/HDAC2 or pCDNA3/HDAC3 expression vector^60^. Cells were harvested for luciferase assays 48 h after transfection. To examine effects of p300 and CBP on GATA6- and Sp1-mediated *Aqp5* transcription, NIH3T3 cells were transfected with -358-*Aqp5*-Luc, together with varying amounts of pCMVβ-p300-myc (R. Eckner, Rutgers University)^61^ or pRc/RSV-m CBP-HA (a gift from R.P. Kwok, University of Michigan)^62^. Cells were harvested for luciferase assays 48 h after transfection.

### WST-1 cell viability assay

Cytotoxicity of SAHA in MLE-15 and NIH3T3 cells was analyzed using WST-1 cell viability assay (#8030, ScienCell Research Laboratories, Carlsbad, CA) according to the manufacturer’s instructions.

### Production of lentivirus in 293 T cells

The lentivirus backbone vector pLK0.1-puro, engineered to express *Hdac3* (TRCN0000039389), *Gata6* shRNA (short hairpin RNA) (TRCN000085589) or non-silencing shRNA control was purchased from Sigma-Aldrich and GE Dharmacon (Lafayette, CO). The lentivirus backbone vector PGIPZ expressing *p300* shRNA (V2LMN_102133 and V2LMN_90586) or non-silencing control shRNA was obtained from GE Dharmacon. Mouse *Gata6* cDNA was cloned into the lentivirus backbone vector pRRL.hCMV.Sin.IRES.GFP^63^. Infectious lentivirus was created by co-transfection of lentivirus vectors expressing *Hdac3*, *Gata6* and *p300* shRNAs and *Gata6* or control vectors with pCMVΔR8.91 and pMD.G into human 293 T cells. The infection mixture was added dropwise to 293 T cells plated on 100-mm culture dishes and incubated at 37 °C overnight. Virus was harvested 48 h or 72 h after transfection, concentrated with PEG-it virus precipitation solution (System Biosciences, Mountain View, CA) and titered with HIV p24 ELISA (Cell Biolabs, San Diego, CA).

### Western analysis

Proteins were resolved by SDS-PAGE and electrophoretically blotted onto Immun-Blot PVDF membranes (Bio-Rad, Hercules, California), followed by blocking in 5% nonfat milk. Primary antibodies (Abs) used included rabbit polyclonal anti-AQP5 (#AQP005, Alomone Labs, Jerusalem, Israel), -p300 (#SC584, Santa Cruz Biotechnology, Dallas, Texas), -HDAC2 (#SC7899, Santa Cruz Biotechnology) and -HDAC3 (SC11417, Santa Cruz Biotechnology). Protein was normalized to eukaryotic initiation factor 2α (eIF-2α), β-actin or GAPDH using rabbit anti-eIF2α (#11386, Santa Cruz Biotechnology) polyclonal Ab and mouse anti-β-actin (LMAB-C4, Seven Hills, Cincinnati, OH) and anti-GAPDH (#AM4300, Applied Biosystems, Foster City, CA) monoclonal Abs, respectively. Blots were incubated with horseradish peroxidase-linked anti-IgG conjugates (Promega) for 45 min at room temperature (RT). Complexes were visualized by enhanced chemiluminescence (ECL) (Thermo Scientific, Waltham, MA) with a FluorChem 8900 Imaging System (Alpha Innotech/Cell Biosciences, Santa Clara, CA).

### Hdac2, Hdac3, Gata6 and p300 knockdown

One day after plating, MLE-15 cells grown on 24-well-plates were transduced with lentivirus expressing *Hdac3* or non-silencing shRNA at MOI = 2. Two days later, cells were transfected with siRNA targeting mouse *Hdac2* (sequence: 5′-GAAUCCGGAUGACUCAUAATT-3′) or control GFP siRNA (Thermo Fisher Scientific) (75 pmoles) using Targefect (Targeting Systems, El Cajon, CA). Protein was harvested for western analysis three days after the second transfection. For *Gata6* and *p300* knockdown, MLE-15 cells grown on 24-well-plates were transduced with lentivirus expressing *Gata6*, *p300* or control shRNA at MOI = 2. Cell lysates were harvested three days after transduction.

### qRT-PCR

To quantify *Gata6*, *Aqp5* and *p300* expression, cDNA was synthesized using SuperScript III reverse transcriptase (Life Technologies, Camarillo, CA) and qRT-PCR was performed using the following primers: *Gata6* forward 5′-ACGCCTCTGCACGCTTTCCC-3′ and reverse 5′-GCCGCCACCTCCACTCACAC-3′; *p300* forward 5′-CCAGCCACCCATGGGAGCAA-3′ and reverse 5′-AGCCAATTCCTTGGGGCTGCT-3′; *Aqp5* forward 5′-CGCTCAGCAACAACACAACACC-3′ and reverse 5′-GACCGACAAGCCAATGGATAAG-3′; *18S* forward 5′-CTTTGGTCGCTCGCTCCTC-3′ and reverse 5′-CTGACCGGGTTGGTTTTGAT-3′ using SYBR-Green PCR with the 7900-HT Fast Real-Time PCR System (Applied Biosystems). The amplification protocol was set as follows: 95 °C denaturation for 10 min followed by 40 cycles of 15-s denaturation at 95 °C and 1 min of annealing/extension at 60 °C.

### Chromatin immunoprecipitation (ChIP)

Chromatin from MLE-15 cells with SAHA treatment, GATA6 overexpression or HDAC3 knockdown as well as corresponding controls, was cross-linked with 1% formaldehyde according to the manufacturer’s instructions (ChIP-IT Express Kit, Active Motif, Carlsbad, CA). For ChIP, ~100 µg of soluble chromatin, 20 µl of protein A/G plus-agarose beads, 20 µl of ChIP dilution buffer (150 mM NaCl, 16.7 mM Tris pH 7.5, 3.3 mM EDTA, 1% Triton-X-100, 0.1% SDS and 0.5% Na-deoxycholate), 1 µl of protease inhibitors and 2 µg of anti-acetyl-histone 3 (H3) (#06-599, EMD Millipore, Billerica, MA) or anti-acetyl-histone 4 (H4) (#39925/39926, Active Motif), or control Ab, were incubated in 200 µl at 4 °C overnight. After washing with a combination of ChIP dilution buffer, radioimmunoprecipitation assay buffer (RIPA) (#R0278, Sigma-Aldrich)/LiCl and Tris-EDTA Buffer (TE), beads were resuspended in 100 µl of 10% Chelex-100 resin (Bio-Rad) and boiled for 10 min, followed by addition of 1 µl of proteinase K and boiling for another 10 min. Following centrifugation, supernatants were collected. DNA fragments encompassing putative enhancer regions homologous to the proximal 358-bp of the rat *Aqp5* promoter were amplified using SYBR-Green PCR (Applied Biosystems, Thermo Fisher Scientific) in a 20 µl reaction volume supplemented with 0.25 µl of 5 Prime HotMaster Taq DNA polymerase (FP2200330, Fisher Scientific) and betaine (1 M final concentration; #B2629, Sigma) with the 7900-HT Fast Real-Time PCR System (Applied Biosystems). Primer pairs were as follows: forward 5′-CGCTGAGCGCACGATGCG-3′ and reverse 5′-CGGCTCCCGCGCCCAGAG-3′. The amplification protocol was set as follows: 96 °C denaturation for 5 min followed by 40 cycles of 1 min denaturation at 96 °C and 80-s of annealing/extension at 70 °C.

### Cross-linking Ab to protein A/G beads

Protein A/G Sepharose beads (30 μl, Santa Cruz Biotechnologies) were incubated with 2 μg of HDAC3 (#SC11417, Santa Cruz Biotechnology), Sp1 (#SC17824, Santa Cruz Biotechnology), GATA6 (#5851, Cell Signaling) Ab or corresponding IgG overnight at 4 °C after equilibration with dilution buffer (0.1 mg/ml bovine serum albumin (BSA) in PBS). After washing with dilution buffer, beads were buffered in 0.2 M triethanolamine (pH 9) and Abs were cross-linked by incubating beads with a final concentration of 6.5 mg/ml of dimethyl pimelimidate (DMP) (#150945, MP Biomedicals, Santa Ana, CA) in 0.2 M triethanolamine (pH 9) for 30 min. Cross-linking was quenched using 0.5 ml quench buffer (50 mM ethanolamine in PBS for 2 hr at room temperature). Excess (unlinked) Ab was removed by washing with 1 M glycine (pH 3). Beads were then washed with RIPA buffer.

### Co-immunoprecipitation (Co-IP)

Total protein lysate (70 µg in 200 µl RIPA buffer) from MLE-15 cells transduced for 48 hours with lentivirus expressing *Hdac3* or non-silencing shRNA was used for each IP. Thirty µl of cross-linked HDAC3, Sp1, GATA6 Ab or corresponding IgG was added to each sample and incubated in a rotator overnight at 4 °C. After washes in RIPA buffer, immunoprecipitated proteins were eluted in Laemmli sample loading buffer and boiled for 15 min. Samples were then subjected to western analysis for HDAC3, GATA6, Sp1 and p300 (#SC 584, Santa Cruz Biotechnology).

### Statistical analysis

Data are shown as mean ± SEM, where (n) is the number of observations. We performed two-way ANOVA, t-tests and z-tests for ratiometric data to determine significance. P < 0.05 was considered significant.
